# The effect of climate change on the resilience of ecosystems with adaptive spatial pattern formation

**DOI:** 10.1111/ele.13449

**Published:** 2020-01-07

**Authors:** Robbin Bastiaansen, Arjen Doelman, Maarten B. Eppinga, Max Rietkerk

**Affiliations:** ^1^ Mathematical Institute Leiden University 2300 RA Leiden The Netherlands; ^2^ Department of Geography University of Zurich 8057 Zurich Switzerland; ^3^ Department of Environmental Sciences Copernicus Institute Utrecht University 3508 TC Utrecht The Netherlands

**Keywords:** adaptability of patterns, critical transitions, desertification, ecosystem resilience, regime shifts, self‐organisation, spatial ecology, spatial patterns

## Abstract

In a rapidly changing world, quantifying ecosystem resilience is an important challenge. Historically, resilience has been defined via models that do not take spatial effects into account. These systems can only adapt via uniform adjustments. In reality, however, the response is not necessarily uniform, and can lead to the formation of (self‐organised) spatial patterns – typically localised vegetation patches. Classical measures of resilience cannot capture the emerging dynamics in spatially self‐organised systems, including transitions between patterned states that have limited impact on ecosystem structure and productivity. We present a framework of interlinked phase portraits that appropriately quantifies the resilience of patterned states, which depends on the number of patches, the distances between them and environmental conditions. We show how classical resilience concepts fail to distinguish between small and large pattern transitions, and find that the variance in interpatch distances provides a suitable indicator for the type of imminent transition. Subsequently, we describe the dependency of ecosystem degradation based on the rate of climatic change: slow change leads to sporadic, large transitions, whereas fast change causes a rapid sequence of smaller transitions. Finally, we discuss how pre‐emptive removal of patches can minimise productivity losses during pattern transitions, constituting a viable conservation strategy.

## Graphical abstract

Classical measures of resilience cannot capture the emerging dynamics in spatially self‐organized, patterned systems, including transitions between patterned states that have limited impact on ecosystem structure and productivity. To complement these classical concepts, we present a framework of interlinked phase portraits that appropriately quantifies the resilience of patterned states, which depends on the number of patches, the distances between them and environmental conditions.

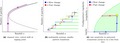

## Introduction

The increasing incidence of extreme climatic events, disease outbreaks and other environmental perturbations have led to a global recognition of the need to conserve ecosystems, and thus to understand the (lack of) resilience of threatened ecosystems (Hodgson *et al.,* 2015; Willis *et al.,* 2018). Classically, resilience has been defined as the capacity of an ecosystem to persist or maintain function in the face of exogenous disturbance (Holling, 1973; Walker *et al.,* 2004). Holling (1973) quantified this capacity as the magnitude of change an ecosystem can withstand: typically small ecosystem changes are manageable, whereas larger changes may lead to critical transitions or catastrophic shifts (Holling, 1973; Scheffer *et al.,* 2001, 2009). Specifically, the paradigm has been highly influential in the development of ecosystem restoration theory (Suding *et al.,* 2004) and the operationalisation of a safe operating space for humanity within the Anthropocene (Rockström *et al.,* 2009; Steffen *et al.,* 2015). However, insights from pattern formation theory have identified system characteristics that may hamper application of classical resilience measures in spatially extended ecosystems.

**Figure 1:**
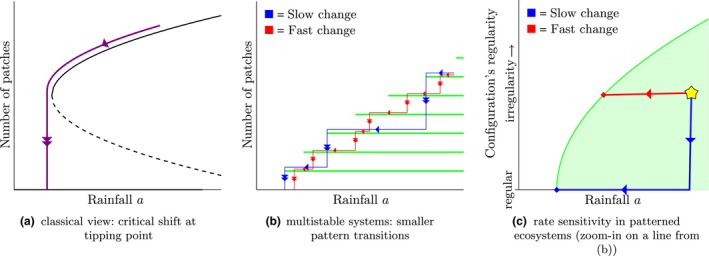
Differences between classical views on ecosystem resilience and resilience in patterned ecosystems. Classically, a change in environmental conditions corresponds to a minor adjustment of the ecosystem state, until changes drive the system over a tipping point and a critical shift occurs (a). In multistable systems, every set of environmental conditions allows for multiple patterned states (b); here, instead of one critical shift, multiple smaller pattern transitions from one patterned state to another occur – that have minor impact on the system's function. Moreover every green line in (b) corresponds to a (fixed) number of patches in the system. However, resilience of a state is determined not only by the number of patches, but also by their configuration. In (c) a zoomed‐in sketch of one such line is given, where the green area indicates feasible configuration types for various levels of the rainfall parameter *a*. It is found that regular configurations – when water is distributed equally among the patches – are the most resilient, and can persist for lower rainfall values than the more irregular configurations. When environmental change drives a system state outside of the feasible region, not all patches can be maintained any longer and, as a consequence, a pattern transition occurs in which some patches wither. How many (and which) patches disappear during such transition again depends on the type of configuration: regular patterns typically lose half their patches (i.e. a period doubling), whereas irregular patterns lose only one (the weakest patch). Moreover – and very importantly in patterned ecosystems – the patches in a pattern are not static; on the contrary, patches slowly try to rearrange themselves into a regular configuration. Hence, under worsening climatic conditions two effects compete: (i) the movement of patches trying to rearrange themselves into a regular configuration and (ii) the shrinking of the feasible region due to climatic change. It depends on the rate of change which effect prevails, and as such two distinct trajectories can be identified (b and c). First, if change is slow (blue in (b and c)), patches have enough time to rearrange themselves into a regular configuration, and stay in that configuration until, for a relatively low rainfall value, a pattern transition occurs in which half of the patches disappear. The process then continues in the same way until the system has degraded into the desert state. Second, if the change is fast (red in (b and c)), patches do not have time to rearrange. Therefore the patterned state leave the feasible region relatively soon, and one patch disappears in the then occurring pattern transition. The process then repeats itself, and patches keep dying out one‐by‐one until the system is captured in the desert state.

Pioneering studies of resilience utilised models that do not take spatial effects into account (Holling, 1973; Noy‐Meir, 1975; May, 1977). Such systems can thus only adapt via spatially uniform adjustments. In reality, an ecosystem's response to environmental change is not necessarily spatially uniform, and can lead to the formation of (self‐organised) spatial patterns in the system (Klausmeier, 1999; von Hardenberg *et al.,* 2001; Rietkerk *et al.,* 2002; Rietkerk & Van de Koppel, 2008) – even in the absence of (driving) spatial inhomogeneities in the environment. In so‐called patterned ecosystems, these patterns emerge when resource scarcity is high and constitute a way to optimise resource usage when uniform coverage can no longer be sustained (Siteur *et al.,* 2014). In this way, patterns already lead to an improved ecosystem resilience. Additionally, a set of environmental conditions (parameters) allows for a wide variety of patterns; that is, these model and real systems have a multistability of patterned states (Bel *et al.,* 2012; Siteur *et al.,* 2014; Bastiaansen *et al.,* 2018b). As a consequence, if environmental changes force a patterned ecosystem to change, a pattern adaptation occurs, causing the system to shift from one patterned state to another – meanwhile limiting the effect on the ecosystem's productivity during the ecosystem shift – see Figure 1. It is difficult to predict the precise pattern adaptations that are going to occur. Previous studies have indicated that this is related to both the severity and rate of the changes in the ecosystem's environment. Depending on these, the pattern adaptations might be minor adjustments, with little effect on the productivity, or larger alterations – or even ecosystem collapses – with stronger effects on the productivity (Siteur *et al.,* 2014).

Patterns are ubiquitous and can be found in variety of ecosystems, including intertidal systems (Van Der Heide *et al.,* 2010; Liu *et al.,* 2014), boreal (Eppinga *et al.,* 2010) and tropical peatlands (Larsen & Harvey, 2010), and freshwater marshes (Koppel & Crain, 2006). However, dryland ecosystems are often used as the prototypical example (Rietkerk & Van de Koppel, 2008) – as we will do in this paper. In these resource limited areas, patterns are self‐organised (Rietkerk *et al.,* 2004; Rietkerk & Van de Koppel, 2008) and reported patterns include gaps, mazes, spots and bands (von Hardenberg *et al.,* 2001; Rietkerk *et al.,* 2002; Rietkerk & Van de Koppel, 2008). Here, (further) decreases in resource availability lead to pattern adaptations, triggering shifts from one patterned state to another – or to a desert state without vegetation (Rietkerk *et al.,* 1997; Siteur *et al.,* 2014). In this context, the difference between a gradual and a catastrophic shift has large impact on biomass in these systems – and on the many livelihoods that depend on these ecosystems (Vetter, 2009; Sissoko *et al.,* 2011). Thus, it is quite urgent to develop more accurate measures of resilience that are able to distinguish between gradual and non‐gradual shifts. Recent findings suggest that such measures should capture the adaptability of patterned dryland ecosystems under various (changing) environmental conditions – which can be utilised to minimise biomass losses during pattern transitions.

To adequately assess ecosystem resilience two questions are important: (1) how much change in environmental conditions (considering both the magnitude and rate of change) will trigger an ecosystem transition; and (2) what is the state the ecosystem will transition to. Classical resilience theory has been developed based on the theory of (simple) ordinary differential equations, which only have a limited amount of (alternative) stable states corresponding to a vastly different system structure and behaviour. In these systems, answering the former question – which can be done by means of classical indicators (e.g. critical slowing down, increasing temporal variance, increasing auto‐correlation; see e.g. table 1 in Dakos *et al. *(2012) for a comprehensive list) – thus automatically answers the second question. However, for multistable systems – including patterned ecosystems – this is no longer true; for these systems, classical indicators can still answer the first question, but answering the second question requires novel indicators that properly account for the aforementioned pattern adaptations to distinguish gradual and catastrophic ecosystem shifts. Hence, for systems that exhibit multistability and rate‐dependent pattern adaptations, new methods are needed to quantify ecosystem resilience that are based on models that account for spatial effects – such as models consisting of partial differential equations.

In this paper, we present a mathematical method to describe the adaptability and resilience of patterned ecosystems. This method considers so‐called ‘strongly localised’ patterns that constitute several patches with vegetation, and bare soil between them – the typical states for systems that experience severe resource scarcity. As an archetypical example, we use a (relatively) simplified ecosystem model of reaction‐diffusion type and use mathematical techniques from Bastiaansen & Doelman (2019) to capture the essential behaviour of these patterns in the model. These give insights in the spatial (re)arrangement of patches and the pattern adaptation that occurs when resources can no longer sustain a specific patterned state. This also shows how these adaptations lead to an overall improved ecosystem resilience. Moreover this application of mathematical techniques provides a framework to study the degradation process of ecosystems based on the rate of climate change, making a distinction between gradual and catastrophic ecosystems shifts possible. Finally, we employ numerical simulations to assess several ecosystem conservation strategies that aim to minimise biomass losses during this degradation process.

## Theoretical framework

To describe the dynamics of vegetation patterns in semi‐arid climates, a large variety of theoretical models have been created – mostly reaction‐diffusion models (Klausmeier, 1999; von Hardenberg *et al.,* 2001; Rietkerk *et al.,* 2002; Gilad *et al.,* 2004). These models describe the interaction between the available water and the vegetation in these ecosystems. A general feature in these models is the difference in timescales between the spreading of water (minutes to hours) and of vegetation (months to years). This separation of (space‐time)scales has been identified as the driving mechanism behind the formation of spatially separated patterns (Turing, 1953; Rietkerk & Van de Koppel, 2008); it is responsible for the following two feedback loops that cause pattern formation: (1) a long‐range negative feedback loop of vegetation on itself, via modulation of resource availability and (2) a short‐range positive feedback loop of vegetation on itself, via an increase in the soil's permeability. Ecosystem models show that these coupled feedback loops can generate many sorts of patterns, such as bands, gaps and mazes (Klausmeier, 1999; von Hardenberg *et al.,* 2001; Rietkerk *et al.,* 2002). Importantly, these patterns are not only observed in models, but have also been commonly observed in dryland ecosystems around the globe (von Hardenberg *et al.,* 2001; Rietkerk *et al.,* 2002; Deblauwe *et al.,* 2011; Gandhi *et al.,* 2018; Bastiaansen *et al.,* 2018b).

One of the most well‐studied models – and the archetype considered in this article – is an extended version of Klausmeier's ecosystem model (Klausmeier, 1999). The extended‐Klausmeier model describes water, *w*, and vegetation, *v*, dynamics by a set of two coupled partial differential equations. A dimensional version of this model and the physical meanings of the parameters are given in Box 1. In non‐dimensionalised version it is given by the partial differential equation.

Box 1 **Scalings**

Throughout this paper, a scaled, non‐dimensionalised version of the extended‐Klausmeier model is used. In this box, we relate the results of and assumptions on the scaled model, as presented in the main text, to those of the non‐scaled dimensional model.

**Dimensional extended‐Klausmeier model**

The extended‐Klausmeier model captures the interplay between water (*W*, measured in *mm*) and vegetation (*V*, measured in *kg m* ^−2^) in semi‐arid ecosystems. The dimensional version of this model reads.

(1) $$\left\{\begin{array}{c} \frac{\partial W}{\partial T}=D_{W}\frac{\partial^{2}W}{\partial X^{2}}+\frac{\partial (SW)}{\partial X}+A-LW-RWV^{2};\\ \frac{\partial V}{\partial T}=D_{V}\frac{\partial^{2}V}{\partial X^{2}}-MV+RJWV^{2}. \end{array}\right.$$

Here rainfall is modelled as a constant supply of water, at rate + *A* (measured in *mm year* ^−1^). Water is lost due to evaporation at rate ‐*LW* (where *L* has units *year* ^−1^), and through uptake by vegetation at rate ‐*RWV* ^2^ (with *R* measured in *mm year* ^−1^ kg^−2^). The parameter *J* (in *kg L* ^−1^) models the increase of biomass per unit of water consumed by the vegetation, which leads to a plant reproduction rate + *RJWV* ^2^. Vegetation is lost due to plant mortality at rate ‐*MV* (with *M* in *year* ^−1^). The parameter *S* is the speed at which water flows downhill, which is proportional to the terrain's slope gradient (and measured in *m year* ^−1^). Finally, *D_W_* respectively *D_V_* are the diffusion coefficients of water respectively vegetation (both carrying units *m* ^2^ *year* ^−1^). See also Siteur *et al. *(2014).

In this paper, a non‐dimensionalised version of the extended‐Klausmeier, (6), is used. This one can be obtained from the dimensional version, (1), by the following set of scalings:

(2) $$\begin{array}{cccc} w=\frac{\sqrt{R}J}{\sqrt{L}}W & v=\frac{\sqrt{R}}{\sqrt{L}}V & x=\frac{\sqrt{L}}{\sqrt{D_{W}}}X & t=LT \end{array}\;$$

(3) $$\begin{array}{cccc} a=\frac{\sqrt{R}J}{L\sqrt{L}}A & m=\frac{1}{L}M & s=\frac{1}{\sqrt{LD_{W}}}S & D=\frac{\sqrt{D_{V}}}{\sqrt{D_{W}}} \end{array}$$

**Dimensional pulse location differential equation**

The dimensionless pulse location differential eqn, (10), can be brought back to the original, physical parameters and variables with the use of the scalings (2)–(3). We denote the location of the patches in the original variables as $P_{1},\ldots ,P_{N}$, where $P_{j}=\frac{\sqrt{D_{W}}}{\sqrt{L}}p_{j}\left(j=1,\ldots ,N\right)$. Then, their evolution is given by

(4) $$\frac{dP_{j}}{\mathrm{dt}}\;=\;\alpha \left[W_{X}{\left(P_{j}^{+}\right)}^{2}-W_{X}{\left(P_{j}^{-}\right)}^{2}\right]\;\text{where}\;\alpha \;=\;\sqrt{D_{V}}D_{W}\frac{R\sqrt{R}J^{3}}{L\sqrt{L}}\frac{A}{\sqrt{M}M}.$$

**Assumption that patches disappear on a fast timescale**

From Bastiaansen & Doelman (2019) it is known that the eigenvalues λ associated to the disappearance of vegetation patches are of order *m*, that is, λ=O(m). Hence, patches disappear roughly at rate *m*. So patches disappear very fast compared to the change in aridity if $\left|\frac{\mathrm{da}}{\mathrm{dt}}\right|\ll m$. In the original physical parameters this gives the assumption $\left|\frac{\mathrm{dA}}{\mathrm{dT}}\right|\ll \frac{L\sqrt{L}}{\sqrt{R}J}M$. This quantity can be found based on estimates on the size of parameters from Klausmeier (1999); Siteur *et al. *(2014). From this, it is revealed that patches (in reality) disappear on a fast timescale – and satisfy the mentioned assumption – if the mean annual rainfall does not change faster than several tens of *mm year* ^−2^.

**Distinction between slow and fast climate change**

In this article, the difference between slow and fast climate change is determined by which of the two effects prevails: (i) the dynamics on $M_{N}$ according to the pulse location differential eqn (10) or (ii) the shrinking of the feasible part of $M_{N}$. For the purposes of this article, we refer to climate change as slow if the first effect happens on a (much) faster timescale compared to the second effect. With a proper rescaling as in Bastiaansen & Doelman (2019), it can be determined that (i) happens on a timescale of order $\frac{Da^{2}}{m\sqrt{m}}$.

Pinpointing the precise evolution of the boundary of the feasible part of $M_{N}$ is a complicated, technical procedure; however, in Bastiaansen & Doelman (2019) it is derived that this boundary has to satisfy an equation of the form.

(5) $$\frac{f(B(t))}{a{\left(t\right)}^{2}}=C,$$

where *B*(*t*) represents the boundary of the feasible part of $M_{N}$, *f*(*B*) some function and *C* a constant that is time‐independent. Differentiation with respect to *t* of this condition reveals that changes in the boundary *B*(*t*) – that is, process (ii) – occurs on a timescale of $\left|\frac{\mathrm{da}}{\mathrm{dt}}\right|/a$.

Thus, from this we infer that climate change is slow if $\left|\frac{\mathrm{da}}{\mathrm{dt}}\right|\ll \frac{Da^{3}}{m\sqrt{m}}$. Scaling this back to the original, physical parameters this conditions becomes $\left|\frac{\mathrm{dA}}{\mathrm{dT}}\right|\ll \frac{\sqrt{D_{V}}}{\sqrt{D_{W}}}\frac{RJ^{2}}{\sqrt{L}}\frac{A^{3}}{M\sqrt{M}}$. Based on estimates from Klausmeier (1999); Siteur *et al. *(2014) this gives a quantified distinction between slow and fast climate change: if the mean annual rainfall decreases (much) slower than several *mm year* ^‐2^ the change is slow; otherwise it is fast.

(6) $$\left\{\begin{array}{c} \frac{\partial w}{\partial t}=\frac{\partial^{2}w}{\partial x^{2}}+\frac{\partial (sw)}{\partial x}+a-w-wv^{2};\\ \frac{\partial v}{\partial t}=D^{2}\frac{\partial^{2}v}{\partial x^{2}}-mv+wv^{2}. \end{array}\right.$$

In these equations, movement of water is modelled as a combined effect of diffusion $\left(\frac{\partial^{2}w}{\partial x^{2}}\right)$ and advection $\left(\frac{\partial (sw)}{\partial x}\right)$. The latter is due to the gradients of the system's topography, which is proportional to *s*(*x*), the slope parameter. Dispersal of vegetation is described by diffusion $\left(\frac{\partial^{2}v}{\partial x^{2}}\right)$. The parameter *D* measures the ratio between the diffusion rate of vegetation and the diffusion rate of water and is small because of the separation of scales. The reaction terms give the change in water as a combination of rainfall (+*a*), evaporation (−*w*) and the water uptake by vegetation (−*wv* ^2^). Vegetation biomass changes because of mortality (−*mv*) and plant growth (+*wv* ^2^). For simplicity, in this article we restrict the model to one spatial dimension and focus on constantly sloped terrains, that is, s(x)≡s. Note that general behaviour observed in the 1D model typically corresponds well to the behaviour of 2D patterns (Siero *et al.,* 2015).

As eqn (6) includes the spatial processes of diffusion, advection and dispersal, it is clear that the model system not only describes changes in water availability and vegetation over time, but also changes in the system components in space. Because of this, it is possible to study the spatial structures – patterns – in an ecosystem. However, understanding the behaviour of a partial differential equation is much harder than that of a model without spatial effects, such as models that consist of ordinary differential equations. The dynamics of the latter can be captured in phase portraits (Holling, 1973), which describe the behaviour of the system based on the components' current value. Therefore the phase portrait of ordinary differential equations has as many dimensions as the number of state variables in the model; in simple models (with only one to three variables) it is often possible to draw the complete phase portrait. The concept of phase portraits can be extended to partial differential equations. Because of the spatial variation in these models, the phase portraits in these cases are always infinite‐dimensional, regardless of the number of state variables. Therefore it is, in general, practically impossible to visualise the (whole) phase portraits associated to partial differential equations.

Interestingly, there are mathematical techniques that can be used to understand the phase portraits of partial differential equations. When there is a large separation of scales – like in dryland ecosystems – it is possible to get a grip on the infinite‐dimensional phase portrait. In these situations, vegetation is strongly localised and patterns consist of one or several (confined) patches of vegetation and bare soil elsewhere; moreover, under these constraints, a patch can be fully characterised by its centre, and the collection of all of these locations thus fully defines the system state. Therefore, the evolution of the system can be understood completely by following the evolution of the patch locations. That is, for these patterns it is possible to split the infinite‐dimensional phase portrait into several finite‐dimensional phase portraits that are linked to each other (Promislow, 2002; Doelman & Kaper, 2003; Kolokolnikov *et al.,* 2005; Bellsky *et al.,* 2013). In the case of dryland ecosystem models, each of these finite‐dimensional phase portraits describes how a finite number of vegetation patches behaves according to the model – which can be captured in ordinary differential equations. The different phase portraits correspond to a different number of vegetation patches; so one phase portrait describes the behaviour of a system containing one vegetation patch, another phase portrait that of a system with two vegetation patches and so on. All of these phase portraits are interlinked via the behaviour of the full ecosystem model, as is detailed below. Here we do not present a full (mathematical) treatment of the model; we refer to Bastiaansen & Doelman (2019) for that. Instead, in this paper we focus on the implications for system responses for realistic parameter values (according to Siteur *et al. *(2014); Klausmeier (1999)).

### Migration of vegetation patches

For the extended‐Klausmeier model this reduction has been performed in Bastiaansen & Doelman (2019) – a short outline of the mathematical procedure can be found in Box 2. We define *M_N_* as the phase portrait of a system with *N* vegetation patches. Denoting the location of these patches by *p*_1_ *,…, p_N_*, their location changes according to the pulse location differential equation (see Box 1 for a rescaling back to the original dimensional variables).

Box 2 **Overview of derivation of PDE to ODE reduction**

For the interested reader, we present a short outline of the performed reduction from the full partial differential equation (PDE) to an ordinary differential equation (ODE) stipulating the movement of vegetation patches. For a full mathematical treatment of this reduction, including all technical results and assumptions, we refer to Bastiaansen & Doelman (2019); Chen & Ward (2009); Bellsky *et al. *(2013). This reduction can only be performed on so‐called localised patterns (that arise under relatively arid conditions in the model); that is, it is assumed that vegetation is only present at some localised regions – the patch locations. At these locations, the vegetation takes up water such that in the soil there is not much water left. In between patches, there is no vegetation, and the incoming water via precipitation will either evaporate or flow to a vegetation patch. An equation describing the movement of the patches can now be found by zooming in on both type of regions – within patches and in between patches – and connecting them to each other at the edge of the regions. Typically, this requires additional scalings in one or both type of regions. Here we illustrate this process for the extended‐Klausmeier model. For other models the general idea is the same, although explicit expressions may vary.

First, zooming in on a patch location can be done by assuming water dynamics does not play an important role here, and vegetation changes only slowly over time (this is called a `quasi‐stationary' approach in the mathematical literature). Hence, as an approximation, one can set $\frac{\partial v}{\partial t}=0$ in (6) and assume *w* is small and approximately constant in a patch. Thus, *v* can be obtained from the equation.

(7) $$\frac{\partial v}{\partial t}=D^{2}\frac{\partial^{2}v}{\partial x^{2}}-mv+wv^{2}=0,$$

Defining the centre of the *j*‐th patch (at time *t*) as *p_j_*(*t*), it is readily checked that this equation is solved by.

(8) $$v_{j}\left(x,t\right)=\frac{3}{2}\frac{m}{w_{j}\left(t\right)}\;\text{sech}{\left(\frac{\sqrt{m}}{D}\frac{x-p_{j}\left(t\right)}{2}\right)}^{2},$$

where the subscript *j* denotes that this stipulates the concentration of vegetation around patch *j*, and *w_j_*(*t*) denotes the small, constant amount of available water at this patch location (at time *t*).

The only dynamical components of eqn (8) are the change in patch location over time, and the (small) amount of available water within this (moving) patch. Thus, eqn (8) shows that the variation of vegetation in space and time can be described by *p_j_*(*t*) and *w_j_*(*t*). Hence, substituting (8) into the full PDE, (1), opens up the possibility to reduce this PDE equation into an ODE equation describing the movement of the vegetation patch. This leads to the derivation of the patch‐location ODE (10). We refrain from giving the details of this process here, as they are quite technical and require a certain level of profundity of mathematical `asymptotic analysis'; details can be found in Bastiaansen & Doelman (2019).

Second, zooming in on the region between patches can be done by setting *v* = 0 in (6) (as no vegetation is present there). Moreover as water moves relatively quickly, it configures itself into an equilibrium state on a fast timescale – compared to the timescale on which the patches migrate. Hence, one can additionally set $\frac{\partial w}{\partial t}=0$. Then, in between patches, the water, *w*, satisfies the relatively simple equation.

(9) $$0=\frac{\partial^{2}w}{\partial x}+\frac{\partial (sw)}{\partial x}+a-w$$

accompanied with boundary conditions at the patch locations stipulating (almost) no water is present there.

So, to determine the direction (and speed) of a moving patch, one first solves (10) between patches. From that solution, the derivatives $w_{x}\left(p_{j}^{+}\right)$ and $w_{x}\left(p_{j}^{-}\right)$ of water at both sides of the patches can be readily obtained. In turn, these expressions must be substituted into the patch‐location ODE (10), which then gives the patch movement (at the current time). Doing this recursively allows to track the patch movement over time.

We note here that the patch‐location ODE (10) does not capture all the dynamics of the PDE; as explained in the main text, in particular it does not check whether a configuration is feasible, that is, if there are enough resources available for each vegetation patch to survive. Feasibility of a given patch configuration can be checked by a linear stability analysis of the PDE system, as described in Bastiaansen & Doelman (2019). In short, this method computes the eigenvalues and eigenfunctions corresponding to the patch configuration; only if all eigenvalues are negative, the patch configuration is feasible.

(10) $$\frac{dp_{j}}{\mathrm{dt}}=\frac{D}{m\sqrt{m}}\left[w_{x}{\left(p_{j}^{+}\right)}^{2}-w_{x}{\left(p_{j}^{-}\right)}^{2}\right],$$

where $w_{x}\left(p_{j}^{\pm }\right)$ is the water gradient at respectively the right and the left side of the vegetation patch; thus, to solve this ordinary differential equation one first needs to find these water gradients (which, in turn, are influenced by the locations of all vegetation patches) – see Box 2 for more information on how to solve this type of equations. According to this ordinary differential equation, vegetation patches move towards locations where most water is available, which is in line with early hypotheses about pattern formation in these systems (Thiery *et al.,* 1995). Because the movement of vegetation will change water availability over a long range, neighbouring patches will be affected by this movement – that is, vegetation patches influence each other indirectly via this mechanism. From the pulse location differential equation it is clear that a vegetation patch will no longer move (that is, $\frac{dp_{j}}{\mathrm{dt}}=0$) when the water gradient on its right is equal in size to the water gradient on its left (that is, $w_{x}{\left(p_{j}^{+}\right)}^{2}=w_{x}{\left(p_{j}^{-}\right)}^{2}$). These gradients will only be equal if the distance between neighbouring patches is the same. Therefore, on constantly sloped terrains, vegetation patches tend to distribute themselves regularly over the available space (Bastiaansen & Doelman, 2019). Thus, the phase portrait $M_{N}$ has one (attracting) equilibrium: the configuration in which the *N* vegetation patches are regularly distributed. Moreover for more complex topographies – that fall outside the scope of this article – the pulse location differential equation can explain many of the (from a simple model's perspective counter‐intuitive) observations like downhill migration of vegetation patterns (Bastiaansen *et al.,* 2018a; Bastiaansen & Doelman, 2019).

### Disappearance of vegetation patches

The pulse location differential equation does not fully capture the model dynamics; eqn 10 describes the behaviour of the system on the timescales associated with movement of patches. However, the full model also includes processes that happen on a faster timescale. Specifically, patches disappear when they can no longer acquire sufficient resources for persistence; this disappearance of patches occurs on a much faster timescale and therefore needs to be considered separately. That is, it needs to be checked whether there are enough resources available for a vegetation patch to persist. Because of the competition between vegetation patches, there is only enough water available for a patch when other patches are sufficiently far away (precisely what ‘sufficiently far’ entails, depends on the rainfall parameter *a*). Therefore, part of the *N*‐patch phase portrait is rendered unfeasible. When the system evolves to a point in the unfeasible region of $M_{N}$, there are not enough resources to maintain all vegetation patches. Once in this region, flow is directed away from $M_{N}$ and a solution ‘drops off’ of $M_{N}$. At this moment a pattern adaptation occurs. As a result some of the patches disappear and the solution ‘lands’ on $M_{M}$ (with *M < N*), the phase portrait that describes the evolution of the remaining patches. How many and which patches disappear during such a pattern transition depends in a complex way on many factors, which are described in the rest of the text.

**Figure 2:**
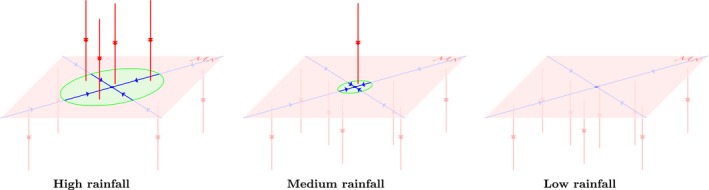
Conceptual illustration of part of the infinite‐dimensional phase portrait of the complete PDE. With an reduction it is possible to capture the dynamics of *N*‐patch solutions in a finite‐dimensional phase portrait, $M_{N}$, which is illustrated as a 2*D* surface in this figure. The blue arrows on this surface indicate the flow of solutions on $M_{N}$ – following these lines corresponds to a change in position of the configuration's vegetation patches. Part of $M_{N}$, indicated in green, consists of feasible patch arrangements, that is, there is enough water available to sustain all the patches. The red part consists of unfeasible solutions. For these patch arrangements there is not enough water available and one or more patches disappear. The feasible part of $M_{N}$ shrinks as the rainfall *a* decreases. For smaller values of *a*, the feasible part of $M_{N}$ can completely disappear; there is a critical value *a_c,N_* – its value depending on the number of pulses – below which $M_{N}$ is completely unfeasible. Precisely at the critical value *a* = *a_c,N_* the regularly distributed patch configuration loses its stability. These critical values are ordered 0 < *a_c,_*_1_ < *a_c,_*_2_ *< … < a_c,_*_*N* − 1_ *< a_c,N_ < a_c,_*_*N* + 1_ *< …*. The red lines in this figure indicate the (fast) dynamics that occur close to $M_{N}$: near the green regions a solution is attracted towards $M_{N}$ (that is, vegetation patches are getting restored in case of disturbance), while a solution is repulsed from it near the red regions of $M_{N}$ – following these lines corresponds to the disappearance of vegetation patches. Ultimately, when a configuration is positioned in the red part of $M_{N}$ one or more vegetation patches die out. The new configuration – with *M* patches (*M < N*) – then follows the dynamics as dictated by the phase portrait $M_{M}$.

A graphical visualisation of part of the full infinite‐dimensional phase portrait for the model can thus be made – as shown in Figure 2. In the figure, the finite‐dimensional phase portrait $M_{N}$, that describes the evolution of *N* vegetation patches – that is, the change in locations of the vegetation patches – is illustrated as a 2D surface. In this plane, the blue arrows indicate how patches rearrange themselves, with the fixed point in the centre denoting the regular configuration. Moreover red arrows indicate the flow perpendicular to $M_{N}$ – that describes the disappearance of vegetation patches. The green part of $M_{N}$ corresponds to the feasible region and the red part to the unfeasible region. Close to the feasible region, the full system is directed towards $M_{N}$, and close to the unfeasible region it is directed away from $M_{N}$ (and towards $M_{M}$ for some *M < N*).

### Effect of decreasing rainfall

The size of the feasible part of $M_{N}$ – that is, the collection of possible configurations with *N* vegetation patches for which there are enough resources available – depends on the amount of resources, that is, on the amount of rainfall *a*. In principle, for relatively arid conditions, the higher the rainfall, the larger the feasible part of $M_{N}$. When the rainfall decreases, this feasible part decreases as well. Ultimately, for some critical rainfall rate *a_c,N_*, the stable part of $M_{N}$ consists of only one patch configuration: the configuration in which all *N* patches are regularly distributed over the available space. Because more resources are necessary to maintain more vegetation patches, these critical values are ordered as 0 < *a_c,_*_1_ < *a_c,_*_2_ *< … < a_c,_*_*N* − 1_ *< a_c,N_ < a_c,_*_*N* + 1_ *< …* Hence, a sustained decrease in rainfall rate may induce a cascade of destabilisations where the destabilisation of a vegetation pattern with *N* patches does not immediately lead to a catastrophic desertification, but it might lead to a new configuration with *M* vegetation patches (*M < N*) that is again feasible until the rainfall is decreased even further. These destabilisations – with only part of the patches disappearing – can thus follow each other, leading to a more subtle and more cascading desertification process (as illustrated in Figure 1).

The boundary between the feasible and unfeasible part of $M_{N}$ describes *when* a *N*‐patch configuration becomes unfeasible and patches disappear. It does not, however, indicate *which* patches disappear. A study of eigenfunctions (those can be seen as generalisations of eigenvectors, but for partial differential equations) has revealed that this depends on the degree of regularity of the *N* patch configuration that becomes unstable (Bastiaansen & Doelman, 2019). If the pattern is a regularly distributed configuration of vegetation patches, multiple patches disappear simultaneously – typically leading to the loss of half of the vegetation patches. On the other hand, if the pattern is irregular, then only one patch disappears: that one patch that has access to the least amount of water, or – equivalent in the mathematical model – that patch that has the least amount of biomass. Thus, the type of pattern adaptation that occurs is related to the regularity of the pattern undergoing the transition: regular patterns experience larger transitions in which many patches die out, while irregular patterns experience smaller transitions in which one patch dies out. This leads to the insight that patterns with the highest resilience (i.e. the regular patterns) undergo the largest ecosystem shifts, while those with lower resilience only experience smaller shifts.

We will now further explore the difference between system responses to slow and fast climate change, based on these findings. Moreover using several numerical simulations it is explored how vegetation biomass can be maximised in these ecosystems via human intervention.

## The difference between slow and fast climate change

The theoretical findings described in the previous section can be combined to determine what happens to vegetation patches under the influence of climate change. For simplicity, and for the purposes of this article, climatic change is characterised only by a change in rainfall, *a*. Here the focus is on scenarios with decreasing rainfall *a*, which leads to the disappearance of vegetation patches due to a lack of resources (in this context: water). Finally, we assume that the disappearance of vegetation patches happens very fast – almost instantaneously – compared to the timescales involved with climate change; that is, we assume $\left|\frac{\mathrm{da}}{\mathrm{dt}}\right|\ll m$ – see Bastiaansen & Doelman (2019); an equivalent condition using dimensional parameters can be found in Box 1.

The system's response to climate change depends on the relative rates of the following two processes. First, the dynamics on $M_{N}$ indicate that patches of vegetation slowly rearrange themselves into a regular pattern. Second, the feasible part of $M_{N}$ shrinks when the rainfall parameter *a* decreases. Thus there are essentially two different scenarios possible, distinguishable by which of these two processes prevails. This distinction can be made based on the rate of change of *a*. When *a* decreases slowly this corresponds to a slow climate change and when *a* decreases quickly this corresponds to a fast climate change. Here ‘slowly’ can be quantified as the requirement $\left|\frac{\mathrm{da}}{\mathrm{dt}}\right|\ll \frac{Da^{2}}{m\sqrt{m}}$; see Box 1 for more information and a translation to the original, physical parameters.

### Slow climate change

**Figure 3:**
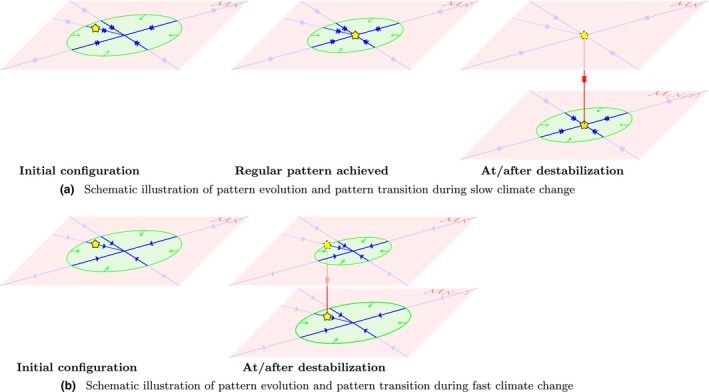
Schematic illustrations of pattern evolution and a pattern transition during respectively slow (a) and fast (b) climate change. Both start from a *N* patch arrangement in the feasible part of $M_{N}$ – indicated by * in the left most illustrations. In both situations the feasible part of $M_{N}$ shrinks because of climate change and simultaneously, the *N* patches migrate, following the dynamics on $M_{N}$. First, when the climate change happens on a slow timescale (a), the feasible region of $M_{N}$ shrinks slowly compared to the patch migration (indicated by the blue double arrows). As an effect, the *N* patch configuration leaves the feasible part only after it has reached the equilibrium position on $M_{N}$, in which the patches are distributed regularly over the domain (a, second panel). When the destabilisation then happens – when *a* = *a_c,N_* – typically half of the vegetation patches are lost. The solution then ‘lands’ on $M_{\left\lfloor N/2\right\rceil }$ and the process of wavelength doubling destabilisations then repeats itself until all patches have disappeared and the system is captured in the desert state (see the blue line in Figure 1b). Second, when the climate change happens on a fast timescale, the feasible region of $M_{N}$ shrinks fast compared to the patch migration (indicated by the green double arrows). As an effect, the *N* ‐patch configuration leaves the feasible part, before it has time to evolve to a regularly distributed configuration (b, second panel). When this happens, the solutions drops off of *M_N_*. Typically one vegetation patch disappears during such transition. The process then continues on $M_{N}$ *_–_*_1_, that consists of all patch arrangements with *N − *1 vegetation patches, until all patches have disappeared and the system is captured in the desert state (see the red line in Figure 1b).

**Figure 4:**
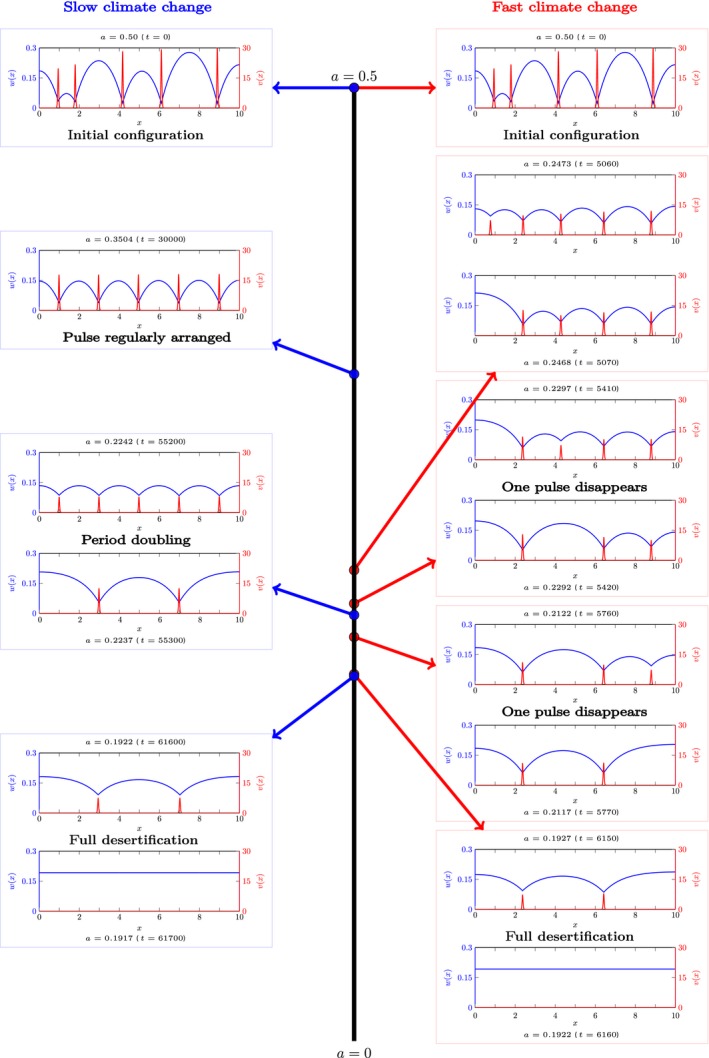
A side by side comparison of simulations of slow (left; blue panels/arrows) and fast (right; red panels/arrows) climate changes. The black line in the middle indicates the value of the rainfall parameter *a*, going from *a* = 0*.*5 (top; start of the simulations) to *a* = 0 (bottom; end of simulations). The insets show the solution at the specified rainfall value, just before and just after a destabilisation. In the fast climate change simulation, patches hardly migrate and they die one by one. In the slow climate change simulation, patches first arrange themselves in a regular pattern, thus increasing their resilience. They can persist in this configuration for lower rainfall values, compared to the irregularly spaced configuration of the fast climate change simulation. However, once the regular pattern becomes unfeasible, a period doubling occurs and multiple patches disappear at once. Note that the simulations end with two pulses disappearing (and not only one), as two consecutive destabilisations happen within the numerical time step used. Simulations made using the numerical method developed in (Bastiaansen & Doelman, 2019), with parameter values *D* = 0*.*01, *m* = 0*.*45, *s* = 0, *a*(*t*) = 0*.*5(1 − *t/T*) where *T* = 10^5^(slow) or *T* = 10^4^(fast).

When *a* decreases slowly, the feasible part of $M_{N}$ also shrinks slowly. Therefore, a generic *N*‐patch configuration (in the feasible part of $M_{N}$) is given enough time to follow the dynamics on $M_{N}$ to the fixed point of this phase portrait – and thus it will rearrange itself into a regularly distributed configuration, with equidistant patches. This regular configuration is then maintained until *a* is decreased below the critical value *a_c,N_* when this state becomes unfeasible. When this happens, typically half of the vegetation patches die out, and a wavelength doubling occurs in the pattern (in case *N* is odd, an approximate wavelength doubling occurs as either $\frac{N+1}{2}$ or $\frac{N-1}{2}$ patches disappear). The process then continues on $M_{\left\lfloor N/2\right\rceil }$, and, with persistent decreases in rainfall, several wavelength doubling destabilisations then occur after each other, until all patches eventually have died out. A schematic illustration of the first part of this process is given in Figure 3a. Moreover a simulation of the full process is presented in Figure 4.

### Fast climate change

In the other scenario, the rainfall *a* decreases on a relatively fast timescale, and therefore the feasible part of $M_{N}$ shrinks fast. This ensures that a (generic, non‐regular) vegetation pattern does not have enough time to follow the flow on phase portrait $M_{N}$ – and the pattern does not become a regularly distributed configuration. Instead, at some moment – when *a > a_c,N_* – the configuration is positioned in the (rapidly growing) unfeasible part of $M_{N}$. Because the pattern is not regularly distributed, generally only one patch disappears: the patch with the least amount of biomass (or, equivalently, access to the least amount of resources). This process then restarts on $M_{N-1}$ (in the feasible region) and patches will keep disappearing one‐by‐one until all patches have disappeared and the system is captured in the desert state. A schematic illustration of the first part of this process is given in figure 3b. Moreover a simulation of the full process is presented in figure 4.

In case of a regular configuration and fast climate change (which could occur for instance when climatic change accelerates), during the first destabilisation – when rainfall *a* drops below *a_c,N_* – a wavelength doubling occurs in which half the patches die out. However, the remaining patches then typically will not have enough time to evolve to a regular configuration and the process continues as explained above – that is, patches disappear one by one.

## Minimising biomass losses under decreasing rainfall

**Figure 5:**
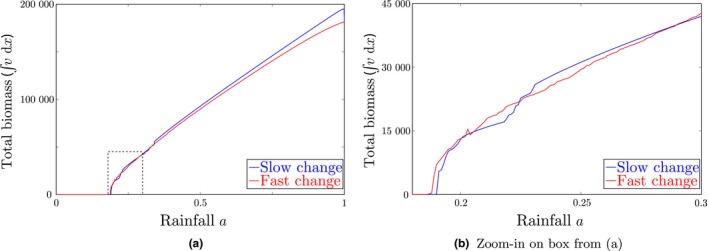
Total biomass, ∫*v*(*x*) *dx*, for simulations of a slow (blue) and a fast (red) climate change. Both simulations started with 88 vegetation patches at the same random locations. During the simulation the rainfall parameter *a* was decreased from *a* = 1 to *a* = 0 in 10^3^ time steps (fast climate change; red) or in 10^6^ time steps (slow climate change; blue). Figure (b) is a zoom‐in on the dashed box in (a) to better show of the difference in pattern transitions occurring during slow and fast climate change.

During the process of desertification, the amount of biomass in the system diminishes. To be able to maximise vegetation biomass, it is therefore paramount to understand how this decline sets in. In Figure 5 the amount of biomass, ∫v(x)dx, is plotted against the rainfall parameter a for systems experiencing fast and slow climate change, respectively. In a slowly changing climate, the pattern first evolves to a regular pattern, yielding higher biomass than an irregular pattern (Figure 5). A direct computation of biomass for all possible two‐pulse patterns (that can be extended to any number of vegetation patches) confirms this (Bastiaansen & Doelman, 2019). In the regular configuration, each vegetation patch has access to the same amount of water, which is the most efficient use of resources for the ecosystem. Hence, given a fixed number of vegetation patches, the most biomass is maintained when these patches are arranged in a regular pattern.

Now that it is clear how vegetation patches should be arranged, the next step is to determine the ideal number of patches in a given system. On the one hand, more patches (and the same amount of resources) leads to more competition between them, and thus might lead to less biomass. On the other hand, more patches means there is a higher vegetation cover, and thus might lead to more biomass. Using formulas from Bastiaansen & Doelman (2019) the amount of biomass per patch can be computed as function of the distance to the patch's neighbours, which in turn yields the amount of biomass per unit length. This procedure indicates that more patches have a positive effect on the total biomass, consistent with recent observations (Bastiaansen *et al.,* 2018b). Hence, to optimise the biomass in a system, in general, the goal is to fit as much vegetation patches as the system possibly can sustain, which should be arranged in a regular fashion to maximise biomass.

Since the critical value *a_c,N_* indicates the lowest value of *a* for which a regular *N* patch configuration can still be maintained, the maximum number of patches in a system can be found as follows: find the largest *N* such that *a_c,N_ < a*, where *a* is the system's rainfall (parameter). Note that the stability of regular vegetation patterns has been studied before (Siteur *et al.,* 2014; Bastiaansen *et al.,* 2018b). These studies have linked the stability of a pattern to its wavenumber (instead of the number of patches as is used throughout this text), where a model‐dependent shape – called a Busse balloon – in the (*wavenumber, parameter*)‐space is made that illustrates all the stable regular patterns. The boundary of this ‘balloon' directly links to the critical values *a_c,N_* and therefore the highest number of patches can be directly inferred from these Busse balloons.

**Figure 6:**
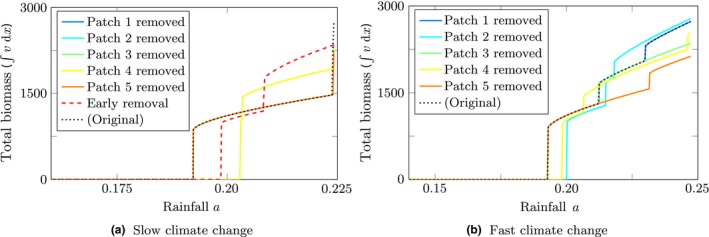
Total biomass, ∫*v*(*x*) *dx*, for simulations of slow (a) and fast (b) climate change, where one of the patches was pre‐emptively removed, just before the first destabilisation of the 5‐patch configuration (of Figure 4). Note that in (a) the colours for removal of patch 1 (dark blue), patch 3 (green) and patch 5 (orange) overlap as do those for removal of patch 2 (light blue) and patch 4 (yellow). Moreover in the case of a slow climate change (a), the effect of an early removal is also given. Here the patch was removed when *a ≈* 0*.*35 such that the remaining patches had ample opportunity to rearrange themselves into a regular 4‐patch configuration; therefore, it does not matter which patch was removed at this early moment as removal of any patch will yield the same results.

**Figure 7:**
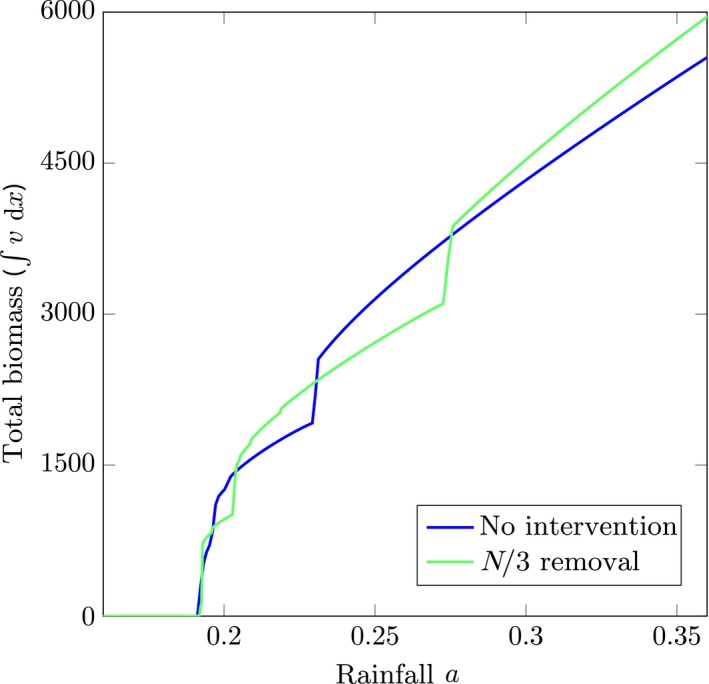
Total biomass, ∫*v*(*x*) *dx*, for two simulations during a slow climate change. In one of the simulations (green) *N/*3 pulses are removed as a precautionary measure right before the initial destabilisation of the regular pattern, while no interventions were made during the other simulation (blue). Both simulations started with *N* = 100 patches in the same regular configuration. During the simulation the rainfall parameter *a* was decreased from *a* = 1 to *a* = 0 in 10^6^ time steps.

However, this does not answer the practical question *how* this can be achieved – and there is no straightforward answer to this either; the long‐term coarsening process of vegetation patches is highly unpredictable and a short‐term optimisation of biomass does not necessarily lead to long‐term optimisation of biomass. For instance, in Figure 5, for high rainfall rates clearly the regular configuration (i.e. the simulation with slow climate change) maintains the highest amount of biomass. Also, this pattern is resilient to changes in rainfall, as it will only undergo a pattern transition when rainfall *a* is close to the critical value *a_c,N_* (with *N* = 88 initially in Figure 5). However, once this threshold is reached, a pattern transition occurs in which half of the patches are lost – thereby skipping many viable patterns with more patches and higher biomass values. Over the same rainfall gradient, the irregular configuration (i.e. the simulation with fast climate change) has been losing patches one by one and therefore does reach those configurations with larger number of patches; although these configurations are irregular, they (can) still have a higher productivity than a regular configuration with fewer patches. As rainfall further decreases, the irregular pattern keeps losing patches and, after enough patches have disappeared, the regular pattern will again have a higher biomass – until it undergoes a new transition in which half of the patches die out and the process repeats itself. This explains the ‘snaking’ in the biomass plots in Figure 5, and also indicates that, because of these processes going on, there is no universal approach to maximising the resilience of a patterned system – as is also illustrated in Figures 6 and 7.

Heuristically, it may seem that a pre‐emptive removal of one patch – close to a destabilisation of the whole configuration – might help to stabilise the others, thereby avoiding a larger pattern transition losing multiple patches. We have carried out this procedure in simulations with fast and slow climate change. In these simulations, we have taken the 5‐patch configuration from Figure 4 and, just before the first destabilisation, we removed one of the patches and then let the simulation continue. The resulting total biomass dynamics are given in Figure 6. Under both fast and slow climate change, removal of any one of the patches, does not lead to any specific consistent benefit. Although some removals lead to higher biomass values initially, these are not consistent for all values of the rainfall parameter *a* and a clear trend does not present itself; it cannot be predicted accurately whether removal of a patch will be beneficial for lower rainfall values; in fact, the removal can even cause the system to degrade into a desert state at a higher rainfall (see Figure 6).

We have also tested an early, *pre‐emptive* removal of one path from a regular *N*‐patch configuration undergoing slow climate change (Figure 6) – where removal of one of the vegetation patches occurred long before destabilisation of the pattern. This pre‐emptive removal allows the system to evolve to a regular (*N − *1)‐patch configuration, which can persist for lower *a*‐values. So the minor sacrifice of one (or a few) vegetation patch(es) increases the resilience of the remaining vegetation in these cases. This method again has a clear short‐term positive effect on the biomass, but the long‐term effects can vary since the next destabilisation happens at higher rainfall values; it might lead to lower amounts of vegetation for lower rainfall values, and, more importantly, final degradation to the desert state might happen at a higher rainfall value. Repeated application of this tactic might remove some of these long‐term drawbacks, but requires intensive monitoring of the system and constant interventions to ensure patches are removed neither too early – when it is not necessary yet – nor too late – when there is not enough time left for the system to rearrange itself into a regular configuration.

Alternatively, if it is not possible to intervene well in advance, it is possible to still minimise the number of lost patches in a destabilisation of a regular *N* ‐patch configuration. These become unfeasible because all patches have access to too little resources. Because of the regularity of the configuration, the system resolves this by the removal of ⌊N/2⌉ patches. For regular configurations, this can also be achieved by the removal of only ⌊N/3⌉ patches: removing every third patch instead of every second patch. This does lead to significant less biomass loss during destabilisation (see Figure 7), but – again – has the drawback that the next destabilisation happens at a higher rainfall value (since $a_{c,\lfloor N/3\rceil }>a_{c,\lfloor N/2\rceil }$). So this process does have a clear short‐term advantage but may have a potential long‐term disadvantage.

## Discussion

Since their discovery on aerial photographs in the 1950s (Macfadyen, 1950a,1950b), spatial vegetation patterns have received considerable attention. Previous hypotheses state that the mere presence of these patterns indicates an ecosystem's proximity to a catastrophic ecosystem shift (Rietkerk *et al.,* 2004; Scheffer *et al.,* 2009). However, these hypotheses implicitly adopted resilience concepts that were derived from non‐spatial models, in which ecosystem resilience is defined as the magnitude of change an ecosystem can cope with (Holling, 1973). These views cannot be extrapolated completely to more refined models that include spatial effects (Siteur *et al.,* 2016); indeed, recent studies have shown that vegetation patterns are more resilient than previously believed (Bel *et al.,* 2012; Siteur *et al.,* 2014; Gowda *et al.,* 2018; Bastiaansen *et al.,* 2018b), and can adapt to changing environmental conditions in various ways (Sherratt, 2013; Siteur *et al.,* 2014; Bastiaansen & Doelman, 2019). The precise extent and severity of these adaptations depends for a part on the magnitude of change, but is also influenced significantly by the rate of change (Siteur *et al.,* 2014, 2016). Therefore, the adaptability of vegetation patterns forms a more comprehensive indicator for the ecosystem's resilience (Bastiaansen *et al.,* 2018b).

In this study, we have provided a framework by which the self‐organising capacities of patterning ecosystems as well as their striking ability to adapt can be understood. We found that, when an ecosystem is not under pressure and there is an abundance of resources, the number of vegetation patches does not change, but their location changes slowly. In this way, each patch optimises its own water uptake, which causes the patches to evolve towards a more regular pattern and enhances the overall pattern's capability to cope with (resource) droughts. In case of such a drought, not all patches can be sustained any more and one, or several, die out because of the lack of resources, increasing the uptake of resources for the remaining vegetation – again increasing the overall resilience of the ecosystem. If this pattern adaptation happens after an ecosystem has evolved into a regular pattern a significant shift in wavelength can be measured, while otherwise only a small change is expected. Hence, a clear difference is found between the degradation of a patterned ecosystem during fast climate change – when patches do not have enough time to optimise their water uptake and disappear one by one – and one during slow climate change – when patches evolve towards a more regular pattern and disappearances occur via more catastrophic wavelength shifts. These interactions between the rate of climate change, the timing of the transition, and the magnitude of the transition are not fully captured by the classical definitions of ecosystem resilience – see also Box 3 for a more elaborate discussion of (classical) engineering resilience and ecological resilience in patterned ecosystems.

Box 3 **Engineering and ecological resilience**

As first proposed in Holling (1996), resilience of (eco)systems can be defined in two different ways, as engineering resilience and as ecological resilience. Engineering resilience deals with fail‐safe design and is measured by the speed at which an equilibrium state is restored after disturbance (also sometimes called the stability). Ecological resilience is related to safe‐fail design and is quantified by the amount of environmental change a system can withstand before it flips to another regime of behaviour – or, more mathematically, another region of stability (or basin of attraction).

Both types of resilience have been studied and explained theoretically using (the theory of) ordinary differential equations. In this article, a framework is presented in which the behaviour of localised patterned states, described via spatially explicit partial differential equations, can be tracked using ordinary differential equations. Hence, the typical methods can be exploited to quantify engineering and ecological resilience of the patterned states – which we illustrate below. However, in these spatially extended systems, both fail to pinpoint critical shifts in the system; that is to say, they do predict (hysteretic) shifts, but these do not (always) have significant impact on the productivity of the system – and certainly do not prelude a change in the driving processes of the ecosystem that is normally associated with a loss of resilience. Thus, as also explained in the main text, these classical methods can be employed to signal an imminent pattern transition, but they need to be supplemented by new methods that can also predict the kind of transition – a minor, non‐critical ecosystem shift or a larger, more critical shift.

**Engineering resilience**

The engineering resilience of a patterned state with *N* patches is given by the speed at which vegetation patches restore their shape after disturbance. This speed is indicative for the distance away from the stability boundary – and does show a critical slowing down. Hence, irregular patterns – that lie close to this boundary – get restored slower and thus have a lower engineering resilience (compared to the more regular configurations that lie more to the middle of the feasible region and restore more quickly). Importantly, as the smallest patch – the one closest to its neighbours – is the first patch to disappear, it is the restorative speed of this patch that is important here – and the one that shows a critical slowing down. For completeness, we note that the (slow) rearrangement of patches is not a good measure for engineering resilience, and also does *not* show critical slowing down close to a pattern transition.

**Ecological resilience**

When considering a regular patterned state with *N* patches, the region of stability is given by all *N* patch configurations which lie in the feasible region (the green regions in Figure 2). Over time, all configurations in this feasible region will slowly rearrange themselves into the regular *N* patch pattern if all parameters stay constant. As explained in the main text, this stability region shrinks as *a* is decreased and fully disappears when *a* drops below the critical value *a_c,N_*. Hence, if *a < a_c,N_* the system flips to another stability region. Thus ecological resilience – the amount of change in rainfall the regular patterned state with *N* patches can withstand – is determined by the difference between the current rainfall value *a* and the critical value *a_c,N_* – that is, by the value *a − a_c,N_*.

Over the years, several empirical studies have been conducted on vegetation patterns in dryland ecosystems. These vary from small‐scale, individual level studies, typically performed using in‐situ measurements, to large‐scale system‐wide studies, typically employing remote‐sensing technology. Due to the increased availability of (historic) aerial imagery and technological advancements, these latter system‐wide studies have become more common and more thorough in the last years (Barbier *et al.,* 2006; Deblauwe *et al.,* 2011). Recently, in‐depth studies on pattern properties corroborated model predictions (Barbier *et al.,* 2014), and empirical evidence was found for multistability in study sites in Somalia (Bastiaansen *et al.,* 2018b). However, empirical studies on the (temporal) behaviour of vegetation patterns – especially on pattern‐to‐pattern transitions – are limited. Most prominently, the persistent decline of patterned vegetation has been highlighted and losses of biomass have been reported in the Sahel region (Wu *et al.,* 2000; Fiorillo *et al.,* 2017; Trichon *et al.,* 2018). Additionally, changes in patterns – pattern transitions – have been found in this region (Barbier *et al.,* 2006; Deblauwe *et al.,* 2011) and explicit examples are given, for instance, in figure 1 in Wu *et al. *(2000), figure 7 in Barbier *et al. *(2006) and figure 6 in Trichon *et al. *(2018). The (averaged) effect of these transitions on the pattern properties has been studied in Wu *et al. *(2000), which reports on an (averaged) increased nearest neighbour distance – that is, a lowered number of vegetated areas. However, to the best of our knowledge, there has not yet been an empirical study on the quantitative changes to the vegetation patterns, linking these to pattern properties such as a pattern's regularity. That said, at this moment, it should be possible to perform such studies, using for example the pattern wavelength and techniques previously used on these systems in Deblauwe *et al. *(2011); Couteron (2001); Barbier *et al. *(2006); Penny *et al. *(2013); Bouvet *et al. *(2018); Bastiaansen *et al. *(2018b).

Interestingly, substantial differences in pattern dynamics have been observed between regions. In the Sahel region, especially near Niamey, almost all patterns have been changing over the last decades or so (Wu *et al.,* 2000; Fiorillo *et al.,* 2017; Trichon *et al.,* 2018). In contrast, patterns have remained largely unchanged within, for example, the Horn of Africa – with the exception of human caused pattern degradation (Gowda *et al.,* 2018). It has been suggested this might be related to a higher grazing pressure in the area near Niamey (Wu *et al.,* 2000; Siero *et al.,* 2019); however, in‐depth studies of sites near livestock concentration points found little evidence for its effect on the vegetation structure (Hiernaux & Gérard, 1999). Alternatively, in light of current work, this difference between dryland ecosystems might also be related to the regularity of the patterns in these regions, which allowed vegetation in the Horn of Africa to self‐organise into more drought‐resilient patterns.

In this way, the regularity of a pattern forms an indicator for both the resilience of the patterned ecosystem as the nature of the coming pattern adaptation. However, it is not possible to predict imminent transitions based (solely) on the pattern's regularity. There is a vast literature on early warning signals for (critical) ecosystem shifts (Scheffer *et al., *,^2009^, ^2001^; Kéfi *et al.,* 2010; Dakos *et al.,* 2011; Scheffer *et al.,* 2012; Kéfi *et al.,* 2014) which can be used on patterned states to predict when a transition is going to happen. For example, critical slowing down is to be expected near a pattern transition; the speed at which a vegetation patch restores its shape after disturbance slows down when a pattern transition is imminent (see also Box 3). However – to stress this once more – the then occurring pattern adaption does not (necessarily) change the ecosystem's structure fundamentally; rather, because of the multistability of these ecosystems, a change from one patterned state to another happens with limited effect on the ecosystem's productivity and resilience. This important difference is caused by the multistability of patterned ecosystems. Typically, studies have focused on systems with a finite amount of stable states – most of the time bistable systems (Kéfi *et al.,* 2010; Scheffer *et al.,* 2012) – where every state corresponds to a vastly different system structure and behaviour. In these systems, a change from one state to another corresponds, almost automatically, to a large ecosystem shift. In multistable systems – where there are a lot of different states – departure from one state to another is typically less dramatic. Hence for patterned ecosystems it is important not only to determine when a transition is imminent, but also what kind of transition is forthcoming. As explained, we expect that the former can be done via the classical methods – which, ultimately, link the size of an ecosystem state's eigenvalues to temporal properties of the ecosystem (Scheffer *et al.,* 2009; Kéfi *et al.,* 2010; Dakos *et al.,* 2011; Scheffer *et al.,* 2012; Kéfi *et al.,* 2014). For the latter, information about the (destabilising) eigenfunctions is needed, which, as our study shows, can be inferred via a characterisation of the (spatial) properties of the pattern itself. Specifically, the model in this paper links the severity of an imminent pattern transition to the regularity of the patch configuration undergoing said transition; other studies have shown a link between the larger critical transitions and the patch‐size distribution (Kéfi *et al.,* 2007; Kéfi *et al.,* 2011; Sheffer *et al.,* 2013).

The current study also inspected several pattern conservation strategies that aim to optimise the amount of biomass in the ecosystem by pre‐emptive removal of part of the vegetation. The basic premise here is that the removal of one patch is beneficial for the remaining vegetation that then has access to more resources, which thus results in an increase in the overall ecosystem's resilience. This can thus prevent an imminent more severe pattern adaptation. This is particularly significant since the desertification process is associated with substantial hysteresis; after the disappearance of a patch, it will not easily reappear – even when climateological circumstances do improve (Trichon *et al.,* 2018). The optimal conservation strategy depends on the rate of change and the form of the pattern, but, in general, it is best to aim for as many patches and arrange them as optimal as possible. Although the right maintenance strategy leads to a short term optimisation of biomass, constant monitoring of the ecosystem is necessary to prevent long term negative effects; if climatological conditions continue to worsen, the next pattern adaptation happens sooner when more patches are present.

The model used in this study is deliberately chosen to be as simple as possible to be able to perform the mathematical techniques. However, the presented results are expected to hold (qualitatively) for more realistic ecosystem models. Nevertheless, because of the simplicity of the model, we have not been able to study all possible mechanisms that might improve an ecosystem's resilience and all strategies that can lead to a higher vegetation productivity. For instance, the used model is not refined enough to study the effect of a partial removal of the vegetation at a patch location (in the model, a patch either disappears completely or is fully restored almost instantaneously). Moreover model studies indicate how the (additional) planting of a pioneering plant species can cause vegetation recolonisation through its positive effect on the infiltration of water (Zelnik & Meron, 2018). Another possibility is to plant trees that provide shade, which reduces the evaporation of water (Millennium Ecosystem Assessment, 2005) – practical successes have already led to a wide‐spread application for agriculture in Sub‐Saharan Africa in the ‘Régénération naturelle assistée' (Farmer Managed Natural Regeneration) program; see for instance Haglund *et al. *(2011) and references within. Of course, in addition to these more recent insights in the inner workings of dryland ecosystems, longstanding agricultural water conservation methods can also be used to improve vegetation productivity in a more direct way (Brauman *et al.,* 2007; Paz‐Kagan *et al.,* 2017) – at least when used with care (Le Maitre *et al.,* 2007).

Although the generic ecosystem model studied in this paper is motivated by observations from arid ecosystems, the main findings do not critically depend on the specific assumptions made to model these systems. Hence, these main findings are relevant for other ecosystems as well. Foremost, in all multistable systems, classical resilience concepts and indicators, based on the theory of ordinary differential equations, fail to indicate the severity of an imminent ecosystem shift. This information thus should be extracted from other (new) indicators. In this paper, we have inspected strongly localised vegetation patterns in dryland ecosystems and found the (ir)regularity of a pattern – that is, the variance in interpatch distances – could provide a suitable indicator for the type and severity of an imminent pattern transition. This general insight also holds for localised patterns in other systems (different indicators are probably necessary for different type of multistable systems). Localised patterns naturally emerge as a consequence of the presence of scale‐dependent feedback loops in (eco)systems (Turing, 1953; Rietkerk & Van de Koppel, 2008). These have been identified in a multitude of patterned ecosystems, including freshwater marshes (Koppel & Crain, 2006), intertidal ecosystems (Van Der Heide *et al.,* 2010) and mussel beds (Liu *et al.,* 2014).

The analytical methods presented in this paper have been developed for a broad range of models of reaction‐diffusion type – including those that possess these scale‐dependent feedback mechanisms and/or phase separation (Alikakos *et al.,* 1991; Promislow, 2002; Doelman & Kaper, 2003; Kolokolnikov *et al.,* 2005; van Heijster *et al.,* 2010; Bellsky *et al.,* 2013). Hence, they can be applied to models of activator‐inhibitor or activator‐depleted substrate type – which are often used to model patterned ecosystems, including the aforementioned ones. That is, their dynamics can be captured by a series of interlinked phase portraits that describe (i) pattern rearrangement and (ii) pattern‐to‐pattern transitions. This reduction needs to be repeated for each new model, as quantitative differences exist from model to model. However, in case of similar ecosystem processes – that is, optimisation of resource distribution – system behaviour is excepted to be qualitatively similar to that described in this paper. Again, patterned ecosystem models often fit this requirement.

Many patterned ecosystems perform important ecosystem roles, but are currently experiencing severe environmental change. Hence, there is an increased demand for reliable early warning signals for these systems. However, these are typically developed within the classical resilience framework, although the characteristics of these ecosystems require a different treatment to accurately account for pattern transitions – as explained in this paper. Therefore, new early warning signals are necessary – based on the insights into the adaptability of patterned ecosystems. These new early warning signals should be developed with both the type of ecosystem as well as their usage in mind; should they signal for all pattern transitions, only major ones or only for full ecosystem collapse? As the present study provides insights into the adaptability and resilience of those ecosystems and their response to changing climatic conditions, it provides a roadmap towards assessing resilience of patterned ecosystems, and thus to the development of early warning signals, using a new combination of analytic tools.

## Authorship

R.B., A.D., M.R. and M.B.E. designed research. R.B. and A.D. performed research. R.B., A.D., M.R. and M.B.E. wrote manuscript.

## Data Accessibility Statement

No new data were used.
